# Skin reaction to capsaicin in patients with systemic lupus erythematosus compared to healthy controls

**DOI:** 10.22088/cjim.12.2.140

**Published:** 2021-03

**Authors:** Maryam Sahebari, Javad Salimi, Peyman Shalchian Tabrizi, Mina Khodabandeh, Nazila Ariaee Nasab, Masoumeh Salari

**Affiliations:** 1 Rheumatic Diseases Research Center, Mashhad University of Medical Sciences, Mashhad, Iran; 2 Allergy Research Center,Quaem Hospital, Mashhad University of Medical Sciences, Mashhad, Iran

**Keywords:** Systemic lupus erythematosus, Substance p, Capsaicin

## Abstract

**Background:**

The interaction between nervous and immune systems has been under investigation. Transient receptor potential vanilloid type 1(TRPV1) is a ligand gated calcium channel expressed by sensory neurons which mediates neurogenic inflammatory response. Substance p which can be released following exposure to capsaicin is a TRPV1 inducer, shown to have altered concentration and function in mice with systemic lupus erythematosus (SLE). We evaluated skin reaction to capsaicin in newly diagnosed and established SLE patients compared to healthy controls.

**Methods:**

Twenty-nine SLE patients (12 newly diagnosed cases under treatment, and 17 established ones, not receiving medications) who referred to rheumatologic disease research center, and 33 healthy subjects of the control group were recruited in this study. A topical solution of capsaicin (0.075%) was applied on the volar forearm during skin test, and time to the tingling sensation, area of induration and area of redness (centimeters^2^) were recorded after 5, 10, and 20 minutes.

**Results:**

The area of redness and area of induration within 15 minutes, time to the tingling sensation (P=0.02), and the overall frequency of tingling sensation (P=0.01) after capsaicin skin test was considerably higher in SLE patients than the healthy controls. Redness, induration and tingling sensation were more frequent but not statistically significant among the established SLE group compared to the newly diagnosed patients.

**Conclusion:**

Since skin reaction to capsaicin is more prominent in SLE patients than the healthy individuals, neurogenic inflammation and the role of P substance should be investigated more in ongoing lupus. Capsaicin test can not predict lupus activity.

Systemic lupus erythematosus is a multisystem inflammatory disease of unknown etiology, in which the failure of self-tolerance leads to autonomic activity of the immune system (1, 2). Different studies have identified the role of genetic components, hormonal and immunologic factors, and environmental agents for the development of this multifactorial disease (3). Recent studies have shown increased levels of neuronal growth factor (NGF) in SLE patients. Accumulation of this substance regulates the peptide receptors responsible for inflammation. NGF, which is actively produced by B-cells, induces T-cell y neuropeptide secretion. The interaction of these signals mediates the function of both the nervous and immune systems, and reveals that a bi-directional circuit exists between these two body systems (4).

Some evidence supports the role of the neuropeptides in developing the different stages of joint inflammation (5). Substance p is one of these neuropeptides, proved to play a significant role in inflammatory pathway activation in autoimmune disease (6). It also transmits pain impulses from the central to the peripheral nervous system (7). 

The nervous system interacts with the joints via primary afferent fibers. Some of these sensory neurons express transient receptor potential vanilloid type 1[trpv1], which is a ligand-gated calcium channel, playing a significant role in the development of neurogenic inflammatory response (8). This receptor is activated via heat, low PH and pungent components such as capsaicin. Antagonists such as capsazepin can block this receptor and alleviate the pain too, but they have adverse effects, such as increased core body temperature. On the other hand, applying the agonists such as capsaicin and its analogues increases intracellular calcium, which leads to the desensitization of the neuron to the stimuli after a while. Furthermore, TRPV1 activation via capsaicin leads to substance p depletion, ultimately resulting in decreased nociception (9). It has been proven that the type of the neuropeptides released from the nerve terminals following TRPV1 activation depends on the type of the stimuli. Capsaicin has been shown to provoke dorsal root ganglion neurons to release substance p from the peripheral and central axons (10). 

Recent studies have suggested that the characteristics of the underlying inflammatory disease also had a pivotal role in the final outcome of stimulating these receptors, as well as the type of the applied vanilloid agonist (11). Considering the fact that the role of TRPV1 in the development of chronic inflammatory pain is obvious, and that dysregulation of the related neuropeptides is accompanied with SLE, we decided to assess the relationship between lupus and the activity of these receptors using capsaicin. This will help to better understand the probable neuropeptide dependent mechanisms underneath lupus manifestations, and it can also evaluate the probable anti-inflammatory and analgesic effects of capsaicin on SLE patients. Therefore, we assessed the skin reaction to capsaicin in SLE patients compared to the healthy subjects, considering different disease parameters.

## Methods

Twenty-nine SLE patients, (twelve newly diagnosed and seventeen established ones) selected from those referred to rheumatic diseases research center between the years 2014 to 2015. The patients of this trial were selected by random sampling method among both the outpatients and hospitalized cases. Subjects were recruited in this study every consecutively by inclusion criteria.

 The newly diagnosed patients were not receiving medications, while the patients with established disease were under treatment. Healthy participants for the control group were selected from among the hospital staff after passing the exclusion criteria, using an accurate interview including past medical, drug history and physical examination.

All patients met the 2012 systemic lupus erythematous international collaborating clinics (SLICC) criteria (12). Due to the limited available publications related to our area of research, and the relatively low frequency of SLE, the patients were enrolled in the study, regardless of their disease stage.

Thirty-three age and gender matched healthy volunteers were also assigned to the control group, after being assessed for exclusion, using physical examination and interview. Informed consent was obtained from the participants. This study was approved by the Ethics Committee (no: 900313), and it was performed in accordance with the Helsinki Declaration, revised 2008. 

To determine the disease activity, patients were considered to have the active disease if their SLE disease activity index 2000 score was greater than 4. Exclusion criteria include: any history of using analgesics specially NSAIDs, antidepressants and tricyclics in the past 2 weeks, history of allergic reactions to chili pepper, asthma, dermatitis, urticaria, any type of hypersensitivity and peripheral neuropathy, current diabetes, drug addiction, pregnancy and breastfeeding. 

Combination therapy for patients with established SLE consisted of prednisolone 5-19mg, hydroxychloroquine or cytotoxics with various doses, depending on the involved organs and disease activity, and the data were documented in the patient’s information checklists. Medication and disease duration were assessed in this study using related checklists and SLEDAI2k (12). Since this investigation has no similar studies, the sample size was determined through the pilot study based on the recruited lupus patients in our study via inclusion criteria.

A round blotting paper with a diameter of 1 cm^2^, stained by 0.1 ml of the capsaicin solution (0.075% concentration) was prepared for each participant. The paper was placed on the anterior forearm, and then covered by a transparent plastic band to prevent evaporation during the test. Time of tingling sensation (itching and burning), which was a subjective symptom, was then recorded. The area of redness at the site of the skin test was uncovered, once after 5 minutes to observe the reaction, and again after 15 minutes in order to measure and record the size of the redness and indurations. First, a certain concentration of capsaicin was applied for the volunteers among members of the research team, and due to the subsequent severe skin reaction, solution concentration was reduced to 1/50 and applied again for them, which was subjectively reported endurable. This reduced concentration of the solution was the same amount that had been reported endurable by the 60 RA patients who participated in previous study of capsaicin skin reaction in RA patients (13). 

We placed a transparent (oily) paper on the skin and traced the shape of the redness area, and the largest diameter of the sketch was recorded. The oily paper was preserved in the patients’ file. To measure the area of the induration 15 mins after exposure to capsaicin, the same method used for reading tuberculin skin test was applied. All the skin test steps were performed by a trained technician. Same measures were used for assessing healthy participants in the control group.

Reading the skin test results, measuring the parameters and determining the disease activity situation and statistical analysis was performed by the medical students, and the faculty staff referred the patients and supervised the skin test steps. Area of the redness marked on the oily paper was measured using a digital scan and adobe photo shop cs5 software. Ultimately, the data were analyzed using SPSS (Version 15) software. T-student (parametric) test was used for normally distributed data, and Mann Whitney (non-parametric) test was used for abnormally distributed data. Results from qualitative variables were reported as percentile ranks. For quantitative variables, results were reported as mean±SD for normally distributed and as median (IQR) for abnormally distributed variables.

## Results

29 SLE patients (12 newly diagnosed and 17 established ones) and 33 healthy participants were recruited in this case-control study and compared after the skin test with capsaicin. All participants were females.

**Comparison of SLE patients with healthy control group: **For control group, median age was 35 years (28-48.5), which was insignificantly different compared to the SLE group (30 years [25.5-41.5 (IQR)]), using Mann Whitney test (P=0.09). Tingling was found in 12 healthy participants (36.4%), whereas 22 SLE patients reported this reaction (75.9%), which was considerably different between these 2 groups (x^2^=13.10, P=0.01). Redness was found in 8 healthy participants (24.2%), while 18 SLE patients reported this reaction (62.1%) found to be considerably different using chi-square test (x^2^=9.07, p=0.01). Two of these healthy participants showed redness within 5 minutes of the exposure, while it was found within 15 minutes of the test among the rest of the participants in both groups. Results from comparing control and SLE groups for redness, induration and time of tingling sensation within 15 minutes of the exposure is summarized in table 1.

**Comparison of patients with established and recently diagnosed SLE patients: **For SLE patients, median age was 31 years (25.25-41.75(IQR)) in the recently diagnosed SLE group and 28 years [24.50-44(IQR)] in the established SLE group, which was not statistically significant (P=0.7). The results comparing recently diagnosed with established SLE patients in demographic features and skin test responsiveness are presented in table 2. As shown in table 2, even though a higher incidence of redness within 15 minutes of the skin test was seen in patients with stablished SLE, it was not statistically considerable. In fact, none of the skin test variables were significantly different between those 2 subgroups of the SLE patients.

**Table 1:** comparing SLE group and control group for redness, induration and time of tingling sensation within 15 minutes of the exposure to capsaicin

**Time to the tingling sensation (minutes)**	**Induration area (cm2)** **Median (IQR)**	Redness area (cm*2) **Median (IQR)**	**Groups**
6.5 (4.75-9)	1.5 (0-3.25)	8.05 (0-12)	SLE patients
3 (2-4)	0	0 (0-24)	Healthy control
p=0.02 z=-2.39	p=0.001 z=-4.84	p=0.01 z=-3.38	p-value

**Association of skin test variables (tingling, redness and induration) with other parameters of the study: **The results in Spearman test did not show any significant difference in time to tingling, redness and induration within first 15 minutes between newly diagnosed and the established SLE group. (pvalue was 0.6, 0.54 and 0.68 respectively). This study showed no significant correlation between redness and induration within 15 minutes, and any of the qualitative and quantitative variables. Tables number 3 and 4 demonstrate these results.

**Table 2:** comparing the variables of the study between the 2 groups of SLE patients; the recently diagnosed and established SLE patients

**Variables**		**SLE patients**			**p-value**
		**Recently diagnosed disease** **N=12**	**established disease** **N=17**	**All** **N=29**	
Age(year)		30 (25. 25-41.75)	28 (24.50-44)	30 (25.5-41. 5)	p=0.71 z=-0.40
BMI(kg^*^/cm^2^) (Mean±SD)		23, 61±4.23	24, 84±3.89	24, 32±3.99	p=0.47 f<0.01
Medications: N(%)	Prednisolone and Hydroxy chloroquine	0 (0)	13 (76.5)	13 (76.5)	
	Prednisolone and Hydroxy chloroquine and Cytotoxic	0 (0)	4 (23.5)	4 (23.5)	
Leukopenia N (%)		1(10.0)	6 (35.3)	7 (25.9)	p= 0.2 x2=2.1
Thrombocytopenia N (%)		2 (20.0)	3 (17.7)	5 (18.5)	p>0.99 x2=0.88
Low C3 N (%)		1 (11.1)	1 (6.7)	2 (8.3)	p=0.99 x2=0.14
Low C4 N (%)		1 (11.1)	2 (13.3)	3 (12.5)	p=0.99 x2=0.14
DsDNA Ratio		1.36(0.30-1.63)	1.23(0.40-1.82)	1.30(0.38-1.76)	p=0.77 Z=-0.3
Proteinuria N (%)		3 (60.0)	2 (22.2)	5 (35.7)	p=0.27 X2=1.1
SLEDAI-2k score		9.35±15.82	8.14±14.94	8.48±15.29	p=0.79 F=0.08
SLEDAI-2k>4		10 (90.9)	15 (88.2)	25 (89.3)	p=0.99 x2=0.05
Cardiac involvement N(%)		2 (16.7)	2 (11.8)	4 (13.8)	p=0.99 x2=0.14
Renal involvement N(%)		2 (16.7)	5 (29.4)	7 (24.1)	p=0.66 x2=0.62
CNS involvement N(%)		2 (16.7)	4 (23.5)	6 (20.7)	p=0.99 x2=0.2
Tingling sensation N(%)		9 (75.0)	13 (76.5)	22 (75.9)	p=0.99 x2=2.9
Time to the tingling sensation(minutes)		6.22±4.99	7.54±3.26	7±4	p=0.46 f=0.7
Positive Redness within 15 minutes		7 (58.3)	11 (64.7)	18 (62.1)	p=0.99 x2=0.12
Induration within 15 minutes(cm^2^)		0 (0-3.47)	2.30 (0-3.25)	1.5 (0-3.25)	p=0.53 z=-0.7
Redness area within 15 minutes(cm^2^)		3.08 (0-11.5)	8.9(0-13.94)	8.05(0-12)	p=0.62 z=-0.55

**Table 3:** relationship between induration and redness within 15minuets of the skin test and qualitative variables of the study

**Variable**		**N**	**Induration (cm** ^2^ **)** **Median (IQR)**	**p-value**	**Area of redness (cm** ^2^ **)** **Median (IQR)**	**p-value**
Leukopenia	yes	7	0 (0-1.5)	p=0.22 z=-1.35	3.97(0-10.56)	p=0.57 z=-0.63
	no	20	2.4 (0-3.5)		6.06 (0-14.46)	
Thrombocytopenia	yes	5	1.5 (0-2.65)	p=0.88 z=-0.2	8.9 (0-10.7)	p=0.79 z=-0.32
	no	22	1.15 (0-3.12)		4.02 (0-13.2)	
Cardiac involvement	yes	4	0 (0-1.5)	p=0.16 z=-1.53	0 (0-7.27)	p=0.12 z=-1.63
	no	25	2.3 (0-3.5)		8.9 (0-13.94)	
Renal involvement	yes	7	0 (0-3.5)	p=0.6 z=-0.56	3.97 (0-20.67)	p=0.94 z=-0.1
	no	22	1.90( 0-3.12)		8.47 (0-11.78)	
CNS involvement	yes	6	0 (0-1.62)	P=0.09 z=1.78	1.98 (0-9.1)	p=0.17 z=-1.44
	no	23	2.5 (0-3.5)		9.89 (0-15.43)	
Medications	no medication	12	0 (0-3.47)	p=0.78 x2=0.42	3.08 (0-11.15)	p=0.71 x2=0.67
	Prednisolone and Hydroxychloroquine	13	2.3 (0-2.9)		9.89 (0-13.94)	
	Prednisolone and Hydroxy chloroquine and Cytotoxic	4	1.75 (0-3.87)		4.02 (0-17.52)	
LowC3	yes	2	1.25 (0-2.5)	p=0.72 z=-0.44	4.94 (0-9.89)	p=0.59 z=0.64
	no	22	1.9 (0-3.5)		6.06 (0-15.71)	
LowC4	yes	3	0 (0-2.5)	p=0.4 z=-0.97	0 (0-9.89)	p=0.27 z=-1.21
	no	21	2.3 (0-3.5)		8.05 (0-15.98)	
Proteinuria	yes	5	0 (0-0.75)	p=0.08 z=-1.92	0 (0-4.45)	p=0.11 z=-1.78
	no	9	2.5 (0-3.75)		9.89 (0-13.49)	

**Table 4:** relationship between induration and redness within 15minuets of the skin test and quantitative variables of the study

**Quantitative variables**	**Time to the tingling sensation(minutes)**	**Induration (cm** ^2^ **)**	**Area of redness (cm** ^2^ **)**
Age(year)	p=0.62 r= -0.11	p=0.26 r=0.22	p=0.67 r=0.08
BMI(kg/cm^2^)	P=0.93 r=0.02	p=0.34 r=0.2	p=0.29 r=0.22
Duration of the disease(months)	p=0.6 r=-0.12	p=0.68 r=0.08	p=0.54 r=0.12
Anti dsDNA /R*	p=0.52 r= -0.16	p=0.14 r=-0.31	p=0.34 r=-0.21
SLEDAI2k		p = 0.13 r = -0.29	p = 0.42 r =-0.16

* : anti dsDNA of patient/normal range of reference laboratory

## Discussion

 In this study, we assessed the skin reaction to capsaicin, as an indicator of the substance p released from sensory terminals in SLE patients, compared to the healthy control subjects. Only a few investigations have been conducted to evaluate the sensitivity to capsaicin in SLE patients. Our study showed that area of redness and area of induration within 15 minutes, time to the tingling sensation, and the overall incidence of tingling sensation after capsaicin skin test were considerably higher in SLE patients, compared to the healthy subjects. Redness, induration and tingling sensation were more frequent among the established SLE group compared to the newly diagnosed patients, which was not statistically significant though. 

Different studies have proven that the interaction between immune and nervous systems in SLE patients, showing that the neurohormonal signals provoke immune system to release inflammatory mediators such as substance p (4). Capsaicin can also provoke neurons to release substance p (6).

Area of redness and induration was significantly larger in SLE patients compared to the control group, while tingling sensation was considerably delayed in SLE group. The main cause for itching and tingling sensation, redness and an induration after exposure to capsaicin is substance p releasing from nerve terminals during inflammation. One study showed that the direct injection of substance p can lead to the significantly increased release of histamine from skin mast cells, while injection and topical application of capsaicin caused very less histamine release in normal human skin, which proved that mast cells and sensory nerves have minimum contact in healthy people (14). In contrast, in lupus patients, each of the disease manifestations may be accompanied with altered concentrations of various neuropeptides (4, 15). 

Serum levels of substance p has been proven to be enhanced in patients with other inflammatory disease, such as osteoarthritis and rheumatoid arthritis (16). To sum up, this issue will remain elusive about SLE until further investigations are performed on peptide concentrations along with the skin tests. It has also been proven that nociceptors are reduced to 63% of the normative value in SLE patients (17), which can explain the delay of tingling sensation observed in SLE patients in our study. Another study performed by a research group in 2006 showed that the mean number of intraepidermal small nerve fibers is reduced in skin samples of SLE patients and the frequency of this reduced density was 13%. These findings had no correlation with disease parameters and parallel active neuropathies (21), which could also be supportive of our results. Our study revealed no significant correlation between disease duration (the newly diagnosed or patients with established lupus) and the skin test parameters. This could suggest the role of substance p in preserving autoimmunity and inducing inflammation in all stages of SLE, from the first acute manifestations to the chronic phase. Moreover, it might suggest that our medical therapies do not inhibit release or activity of substance p. Possible role of other neurotransmitters that can affect the results of these tests should not be neglected though. Our results may suggest that manufacturing medications targeting substance p release or activity, may be beneficial for symptom management of SLE patients.

This study did not show any significant correlation between induration or redness and the disease activity in SLE patients. Prior studies have also shown an association between disease activity and reduced skin nociceptive neurons (17). There were also studies indicating that patients with higher ESR levels and more active disease showed smaller redness areas after the skin test, compared to others (18). Some studies revealed that SLE might occur as a consequence of T-lymphocytes dysfunction and NF-kb translocation (19). On the other hand, it has been proven that capsaicin is capable of inhibiting this translocation (20). Therefore, either of these facts can be suggested as the reason why the skin reaction to capsaicin is not correlated with SLE activity. It can be a new line for future studies. Another study, performed on 60 SLE patients with the mean age of 43.2 in 2006, also showed that the reduced intraepidermal small nerve fibers have no correlation with SLE parameters like disease activity and duration, or demographic variables such as age. Neurotoxicity of cytotoxics and hydroxychloroquine, and their possible adverse effects on sensory nerves should be considered, even though there was no obvious correlation between receiving treatment and skin reaction or denervation neither in our study, nor in the aforementioned research (21).

One of the limitations of this study was stopping the patients’ medications before the skin test was not possible. Although, according to some studies, corticosteroids can only affect the products of inflammation and does not interfere with capsaicin skin test (18). Other limitation is taking immunosuppressive drugs in SLE patients. Since our study revealed no significant correlation between parameters in the newly diagnosed with established subjects, its effect on the skin testing can be ignored. Furthermore, it was not possible for us to evaluate dose dependent skin reactions to capsaicin due to severe reactions reported after exposure to higher concentrations in the pilot study. Therefore, performing similar studies with larger sample sizes and different capsaicin doses are suggested.

In conclusion our study showed that SLE patients have increased sensitivity to capsaicin compared to healthy people. In addition, we showed that disease duration and activity in SLE patients have no significant association with severity of the skin reaction to capsaicin. It seems that substance p plays a role in the inflammation reaction of systemic lupus erythematosus. 
